# An American Text-Book of the Diseases of Children

**Published:** 1894-12

**Authors:** 


*An American Text-book of the Diseases of Children.* By AMERICAN TEACHERS. Edited by LOUIS STARR, M.D., assisted by THOMPSON S. WESTCOTT, M.D. Pp. xiv., 1190. Philadelphia: W. B. SAUNDERS. London: F. J. REBMAN. 1894.

In welcoming this book as a standard addition to the rapidly increasing literature of diseases in children, we admit that the editor has succeeded in making considerable progress in the journey toward the ideal volume he so cleverly describes in his preface, although we can hardly congratulate him on the production of what he calls one "readily-handled volume," for the book is large even to the extent of being cumbersome. Perhaps, however, the issue of this over-large size instead of the publication of the work in two volumes has been wise as the less of two evils.

The work itself is recent in the best sense of the term, not merely as regards the time of its appearance, but is very satisfactory in that it is an exposition of present scientific knowledge and practice. It is the product of the experience of a large number of American physicians and surgeons, each one of whom has had ample clinical experience and understands the subject upon which he writes. The book is a model as regards paper, printing, and pictures; many of the illustrations are as good as can be desired: as examples, we would instance Plate V. (Hutchinson Teeth), Plate VIII. (Scarlet Fever), and Plate XVIII. (Sporadic Cretinism). There are also several excellent coloured illustrations of skin diseases.

It would seem almost invidious in a work so fairly good all round to make distinctions by selecting any articles as of greater merit than others, but still we feel constrained to mention our especial appreciation of "Diseases of the New Born," by Dr. E. P. Davis; "Tuberculosis," by Dr. William Osler; "Hysteria," by Dr. James Hendrie Lloyd, and an extremely useful contribution from the pen of Dr. B. Alexander Randall on "Diseases of the Ear."

The value of the book is considerably enhanced by a very complete index, the labour of preparing which we think can only be appreciated by those who have been engaged in similar work.

Taken as a whole we can strongly recommend this volume as a trustworthy authority upon the subjects of which it treats.

